# Integrated Metabolomics and Transcriptomics Reveal Bitter Compounds and Synthetic Pathways in the Special-Germplasm Bitter-Tasting *Dendrocalamus brandisii*

**DOI:** 10.3390/plants15040560

**Published:** 2026-02-10

**Authors:** Hao Wang, Dejia Yang, Yongchao Ma, Yongmei Wang, Hui Zhan, Shuguang Wang, Juan Li

**Affiliations:** 1 Forest Resources Exploitation and Utilization Engineering Research Center for Grand Health of Yunnan Provincial Universities, College of Biological Science and Food Engineering, Southwest Forestry University, Kunming 650224, China; wanghao2024@swfu.edu.cn (H.W.);; 2 Biological Research and Utilization Innovation Team in Bamboo Resources of Yunnan Province, Southwest Forestry University, Kunming 650224, China; 3 Edible and Medicinal Fungi Research Innovation Team, College of Biological and Food Engineering, Southwest Forestry University, Kunming 650224, China

**Keywords:** *Dendrocalamus brandisii*, bitterness, electronic tongue, 4-hydroxybenzoate, multi-omics integration

## Abstract

Bamboo shoots represent a traditional food in China, with most varieties exhibiting a bitter taste; however, understanding of the compounds responsible for this bitterness remains limited. In this study, shoots of a special-germplasm bitter-tasting *Dendrocalamus brandisii* (Dbs) were investigated, using sweet-tasting *Dendrocalamus brandisii* (Db) shoots as a control. Electronic tongue analysis, broad-target metabolomics, targeted metabolomics, and transcriptomics were employed to identify the metabolites and key genes associated with bitterness in Dbs shoots. Electronic tongue measurements revealed a significant difference in bitterness between the two groups. Human sensory evaluation confirmed that Dbs was perceived as significantly more bitter and less sweet than Db (*p* < 0.01). Nontargeted metabolomics screening identified 43 differential metabolites, 19 of which were upregulated in Dbs. Targeted analysis of these differential metabolites, combined with the BitterDB database and previously reported bitter compounds, suggested that 4-Hydroxybenzoate, gallic acid, epicatechin, tryptophan, histidine, and apigenin may contribute to the bitterness of Dbs. Among these, 4-Hydroxybenzoate showed an approximately 92-fold higher content in Dbs compared to Db. The taste activity values (TAVs) of the identified bitter compounds were calculated; only 4-Hydroxybenzoate exhibited a TAV greater than 1 (14.581), while the TAV of the other compounds were all below 1. Integrating broad-target metabolomics, targeted metabolomics, and TAV analysis, 4-Hydroxybenzoate was inferred to be one of the primary bitter substances. Transcriptomic analysis indicated significant upregulation of key genes in the biosynthetic pathway of 4-Hydroxybenzoate, including *PAL*, *4CL*, and *C4H*. Enzyme activity assays further demonstrated that phenylalanine ammonia-lyase, 4-coumarate-CoA ligase, and cinnamate 4-hydroxylase activities were markedly higher in Dbs than in Db. RT-qPCR validation confirmed that the expression levels of *4CL3*, *4CL4*, *PAL1*, *PAL2*, *PAL3*, *PAL4*, and *PTAL* were significantly elevated in Dbs, consistent with the transcriptomic data. In conclusion, 4-Hydroxybenzoate is proposed as the most likely key compound responsible for the bitterness in Dbs shoots. This study provides valuable insights into the bitterness formation mechanism in this Dbs and offers important information for the improvement of its edible quality.

## 1. Introduction

Bamboo (*Bambusoideae*, Poaceae) represents one of the world’s fastest-growing plant groups, constituting a significant component of forest ecosystems with substantial commercial value and ecological functions, including critical roles in maintaining ecological equilibrium and mitigating soil erosion [1]. China possesses the planet’s most diverse bamboo resources, encompassing approximately 43 genera and over 700 species, with a total forested area of 7.5627 million hectares [2]. This accounts for more than 25% of the global bamboo forest area [3], leading to its designation as the “Bamboo Kingdom” [4].

*Dendrocalamus brandisii*, a large sympodial evergreen bamboo within the Poaceae family, is recognized as one of the world’s three premier edible-shoot bamboos, alongside *Dendrocalamus hamiltonii* and *Dendrocalamus asper* [5]. Its natural distribution spans subtropical and tropical regions of Southeast Asia, including China (predominantly Yunnan Province), Myanmar, Laos, Thailand, and Vietnam [6,7]. Beyond its ecological value, *D. brandisii* holds significant economic importance. Its shoots are highly prized for their nutritional profile, characterized by high levels of amino acids, minerals, vitamins, and dietary fiber, coupled with low sugar and fat content [6,8]. Furthermore, the species is extensively utilized in Yunnan Province for applications including construction and papermaking, owing to its thick-walled, straight, and high-strength culms.

In recent years, there has been a notable shift in consumer dietary preferences toward plant-based diets, with many individuals reducing their consumption of animal-derived foods, particularly meat [9]. This shift, however, faces a primary challenge: the inherently unappealing taste of many plant materials, which limits their broader adoption [10]. As a popular medicinal and edible plant, bamboo shoots exemplify this issue, as they often exhibit undesirable bitterness that significantly compromises their quality, edibility, and widespread utilization in the food industry.

The Dbs was discovered at Southwest Forestry University. Its morphological characteristics such as bamboo poles, nodes, branches, sheaths, leaves, and bamboo shoots are no different from those of Db. However, when tasting its bamboo shoots, a strong bitter taste was found. The primary bitter compounds responsible for this bitterness in bamboo shoots, the mechanism behind this variation in bitterness, whether it is driven by environmental or natural factors, and the economic losses resulting from such variation constitute a highly significant issue worthy of in-depth investigation.

Sensory characteristics, particularly flavor attributes, are among the core drivers influencing consumer dietary decisions. Generally, people prefer sweet and umami foods while exhibiting an innate aversion to bitter-tasting foods [11]. Bitterness is a fundamental taste modality detectable by human taste perception at very low thresholds [10]. Bitter substances primarily include phenols, flavonoids, alkaloids, amino acids and their derived peptides, saponins, inorganic salts, fatty acids, and their oxidation products [12,13]. These compounds are widespread in various plants such as *Momordica charantia*, bamboo shoots, citrus, tea, coffee, lettuce, etc. [14,15,16]. In bamboo shoots, the primary bitterness-contributing compounds of *Bambusa rigida* are flavonoids [17]; tannins, flavonoids, and cyanides are the main bitterness-contributing substances in shoots of *Neosinocalamus affinis* [17]; the bitterness intensity of *Dendrocalamus latiflorus* shoots shows a significant positive correlation with the contents of tannins, phenylalanine, tyrosine, cyanides, and oxalic acid in the shoots [18]; the post-emergence bitterness of *Pleioblastus amarus* shoots may be caused by an increase in cyanide content after the shoots emerge from the soil [19]. The post-emergence bitterness in *Bambusa oldhamii* shoots mainly results from increased arbutin content [20]. However, the specific compounds responsible for the bitterness in shoots of the unique bitter germplasm of *D. brandisii* remain unidentified.

The BitterDB is currently the only freely searchable bitter compound database. It catalogs over 2200 known bitter molecules to assist users in identifying and analyzing bitter substances in food [21]. Metabolomics is a systems biology approach that comprehensively analyzes metabolites within biological systems. It employs various techniques including nuclear magnetic resonance (NMR), mass spectrometry (MS), liquid chromatography-MS (LC-MS), gas chromatography-MS (GC-MS), ultra-performance liquid chromatography (UPLC), high-performance liquid chromatography (HPLC), and LC electrospray ionization tandem mass spectrometry [22]. High-throughput metabolomics has been effectively used to identify bitterness in plants [23,24,25]. This approach can reveal key metabolic pathways involved in bitter compound production by identifying metabolite patterns correlated with bitterness levels. Transcriptomics, based on RNA-seq technology, analyzes genome-wide gene expression. By examining expression levels of metabolism-related genes, this technique identifies key regulatory genes influencing bitter compound synthesis, enabling deeper insights into the genetic and biochemical processes underlying bitterness [26,27]. Collectively, these complementary omics approaches can identify both regulatory mechanisms (gene expression) and metabolic pathways (metabolites) involved in bitter compound biosynthesis. In recent years, integrated transcriptomic and metabolomic analyses have yielded significant advances in unraveling the mechanisms underlying bitter taste formation in *Dendrocalamopsis oldhamii* [20,28], *Pleioblastus amarus* [29], *Dendrocalamus latiflorus* [19], and *Phyllostachys edulis* [24]. However, the bitter taste-related metabolites and regulatory genes in Dbs remain largely uncharacterized, which has hindered the quality improvement of Dbs.

This study utilized bamboo shoots from a *D. brandisii* of specific germplasm (Dbs) as the research focus, with shoots from the conventional sweet *D. brandisii* (Db) serving as the control. E-tongue analysis combined with integrated metabolomic and transcriptomic analyses was conducted on both types of shoots. The objectives were to identify bitterness-associated metabolites in Dbs, elucidate the biosynthetic pathways underlying bitter compound formation, and delineate key genes involved in this process through multi-omics integration. This research deepens the understanding of bitter compound biosynthesis in bamboo shoots, providing a theoretical foundation for breeding improved *D. brandisii* varieties and for bitter-taste research in other plants, thereby enhancing their economic value.

## 2. Results

### 2.1. Electronic Tongue Analysis and Sensory Evaluation of Two Types of Dendrocalamus brandisii Bamboo Shoots

**Figure 1:**
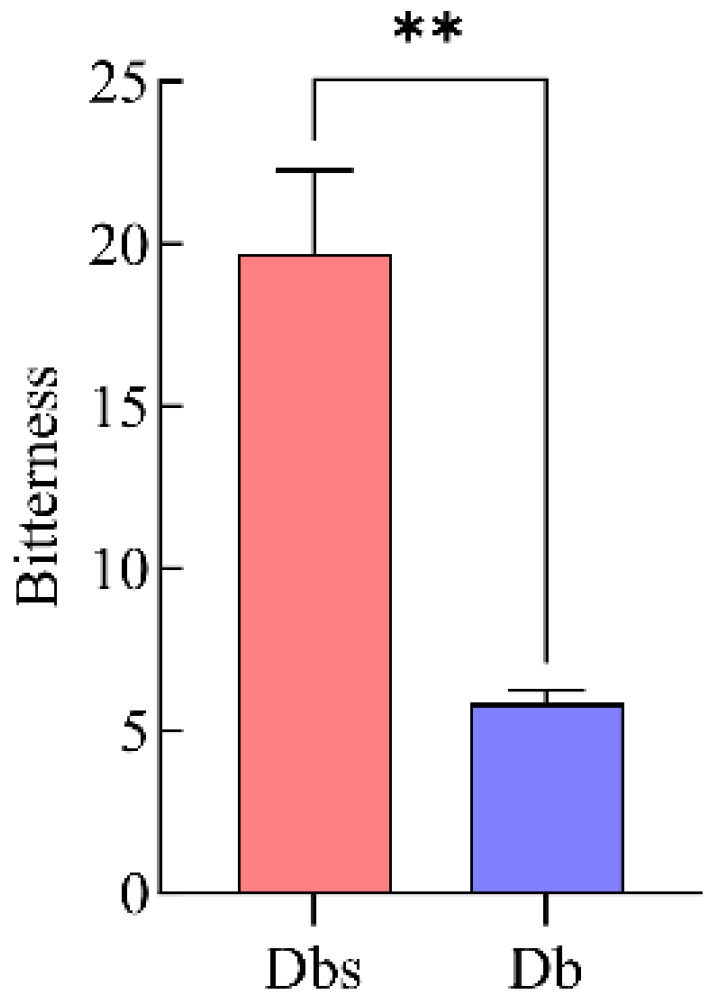
Analysis of the electronic tongue of two kinds of bamboo shoots. Note: Db: *Dendrocalamus brandisii*; Dbs: *Dendrocalamus brandisii* of specific germplasm. ** indicates a significant difference in mean value at the *p* < 0.01 level.

Taste analysis was conducted using an electronic tongue (SA402B model). The readings of the reference solution were set as the zero point. Samples exhibiting output values higher than this baseline were included in the evaluation. The sensor potential responses corresponding to bitterness for the different samples are presented. Electronic tongue analysis of bitterness in the two bamboo shoot samples is shown in Figure 1. According to the bar chart, the difference in bitter aftertaste intensity between the two samples was 13.84. A highly significant difference was observed in their sensor potential responses. Compared to Db bamboo shoots, the Dbs exhibited a higher bitterness intensity. This result is consistent with human sensory evaluation (Table 1). To validate the instrumental taste data at the human perception level, a triangle test was conducted according to ISO 4120:2021. The test confirmed that the overall flavor difference between Db and Dbs shoots is readily perceptible. Out of 12 assessors, 10 correctly identified the odd sample, which exceeds the minimum number required for statistical significance at α = 0.05 (*n* = 9). Subsequent intensity scoring provided quantitative measures of sweetness and bitterness (Table 1). Db shoots received significantly higher sweetness scores (3.8 ± 0.63) compared to Dbs shoots (0.6 ± 0.52) (*p* < 0.01). Conversely, Dbs shoots were rated as significantly more bitter (3.9 ± 0.74) than Db shoots (0.7 ± 0.48) (*p* < 0.01).

### 2.2. Untargeted Metabolomics Analysis of Two Bamboo Shoot Varieties

#### 2.2.1. Sample-Type Grouped Metabolite Quality Control (QC) Analysis

**Figure 2:**
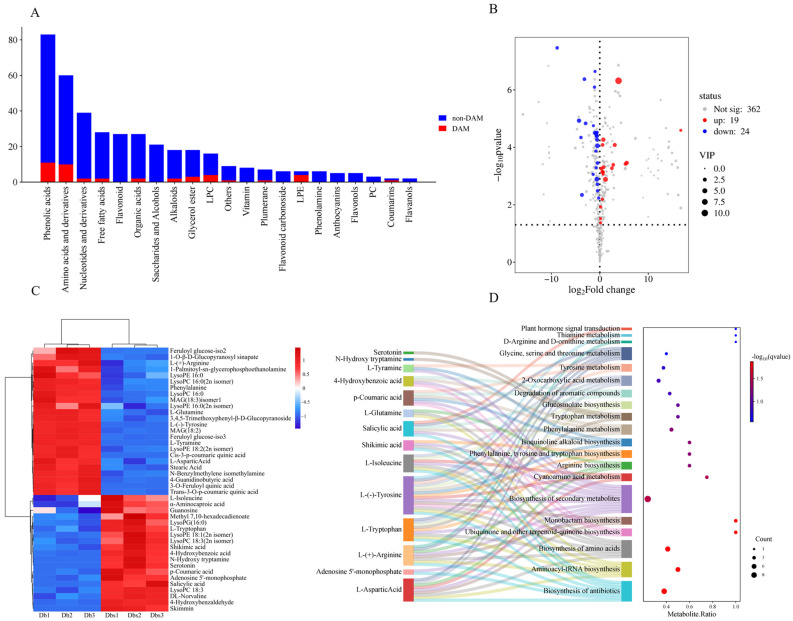
The basic profiles of the non-targeted metabolomics. (**A**). The statistic of DAMs and non-DAMs classes; (**B**). Volcano plot of upregulated and downregulated metabolites; (**C**). Types of differential metabolites between Dbs and Db; (**D**). Metabolic pathways enriched with differential metabolites between Dbs and Db.

Bitterness is a critical determinant of edibility and commercial value in Db bamboo shoots. LC-MS-based Untargeted Metabolomics was employed to tentatively identify bitter compounds in the Dbs shoots. A total of 405 metabolites were detected across both bamboo shoot varieties, primarily categorized as phenolic acids (83 compounds), lipids (72 compounds), amino acids and derivatives (60 compounds), flavonoids (49 compounds), nucleotides and derivatives (39 compounds), others (39 compounds), alkaloids (31 compounds), organic acids (27 compounds), lignans and coumarins (4 compounds), and tannins (1 compound). Detected metabolites were classified into Differentially Abundant Metabolites (DAM) and Non-Differentially Abundant Metabolites (non-DAM) for visualization (Figure 2A). Overlaid Total Ion Chromatograms [20] of replicate QC samples demonstrated consistent retention times and peak intensities for identical metabolites in composite chromatograms, with a high degree of curve overlap. This confirms robust experimental stability and reproducibility, validating the reliability of the metabolomics data.

#### 2.2.2. Differential Metabolite Analysis

Significant differential metabolites between comparative groups were screened by integrating OPLS-DA VIP (Variable Importance in Projection) values and univariate T-test *p*-values, with a significance threshold of VIP ≥ 1 and *p* < 0.05 identifying 43 differential metabolites. Within the Dbs bamboo shoots, 19 metabolites were upregulated and 24 downregulated (Figure 2B). The differential metabolite heatmap (Figure 2C) indicated that upregulated substances included 4 amino acids and derivatives, 4 phenolic acids, 4 lipids, 3 organic acids, 1 lignan/coumarin (skimmin), 2 nucleotides and derivatives, and 1 N-hydroxytryptamine; downregulated substances comprised 6 amino acids and derivatives, 7 phenolic acids, 8 lipids, 2 alkaloids, and 1 organic acid. To determine the source of bitterness in Dbs, 10 bitter compounds were screened through cross-referencing differential metabolites with BitterDB (Table S3), including 5 amino acids and derivatives, 3 phenolic acids, and 1 each from other categories and lipids. Studies by Lifeng Dai et al. demonstrate that bitter compounds primarily derive from polyphenols, alkaloids, saponins, peptides, and amino acids [30,31]. Integrating these findings, we focused on identifying core metabolites potentially involved in Dbs formation from differential amino acids derivatives, phenolic acids, and flavonoids.

To investigate metabolic pathway differences between the two bamboo shoot varieties, functionally annotated significantly differential metabolites were analyzed using the KEGG database. A total of 47 enriched metabolic pathways were identified, with the top four KEGG pathway categories (excluding “Metabolic Pathways”) ranked by differential metabolite abundance being: Secondary Metabolite Biosynthesis (ko01110), Antibiotic Biosynthesis (ko01130), Aminoacyl-tRNA Biosynthesis (ko00970), and Amino Acid Biosynthesis (ko01230). Among the 43 differential metabolites, 18 were enriched across these 47 pathways. Enrichment analysis of the top 20 pathways was performed using −log_10_^(q-value)^ values derived from *p*-values via the Benjamini–Hochberg method (Figure 2D). Analysis of Figure 2D revealed that bitter-tasting amino acids among the screened bitter compounds were primarily enriched in Antibiotic Biosynthesis (ko01130), Aminoacyl-tRNA Biosynthesis (ko00970), Amino Acid Biosynthesis (ko01230), and Secondary Metabolite Biosynthesis (ko01110). Phenolic acid bitter compounds showed dominant enrichment in Ubiquinone and Other Terpenoid-Quinone Biosynthesis (ko00130), Secondary Metabolite Biosynthesis (ko01110), and Phenylalanine Metabolism (ko00360). Notably, the enrichment patterns of bitter compounds differed significantly between Dbs and Db. Subsequent investigations will prioritize these pathways to identify compounds responsible for bitterness in Dbs.

### 2.3. Targeted Metabolomics Analysis of Two Bamboo Shoot Varieties

#### 2.3.1. Targeted Metabolomic Analysis of Phenolic Acids

**Figure 3:**
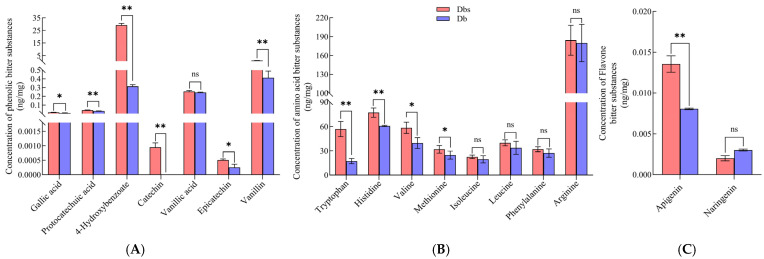
Concentration comparison of various bitter substances between “Dbs” and “Db”. (**A**): Concentration of phenolic acid bitter compounds; (**B**): Concentration of amino acid bitter compounds; (**C**): Concentration of flavonoid bitter compounds. Note: * indicates *p* < 0.05; ** indicates *p* < 0.01; ns indicates no significant difference between the two comparisons.

Targeted metabolomic analysis of phenolic acids in both bamboo shoot varieties (Figure 3A) revealed highly significant differences in 4-Hydroxybenzoate, vanillin, catechin, and protocatechuic acid (*p* < 0.01), significant differences in gallic acid and epicatechin (*p* < 0.05), and no difference in vanillic acid. All 17 bitter compounds exhibited higher concentrations in Dbs than in Db, with 4-Hydroxybenzoate showing a 92-fold increase in Dbs. Although catechins were exclusively detected in Dbs, their concentrations did not reach the threshold for eliciting bitterness perception in humans [32]. Integrating non-targeted metabolomics data with screened bitter compounds, 4-Hydroxybenzoate [33] was identified as a key bitter compound contributing to Dbs bitterness and selected for subsequent investigations.

#### 2.3.2. Targeted Metabolomic Analysis of Amino Acids and Derivatives

Targeted metabolomic analysis of amino acids identified eight bitter-tasting amino acids with elevated concentrations in Dbs compared to Db (Figure 3B), specifically histidine [34], tryptophan [35], valine [36], methionine [37], isoleucine [38], leucine [39], arginine, and phenylalanine [39]. Highly significant differences (*p* < 0.01) were observed for histidine and tryptophan, significant differences (*p* < 0.05) for valine and methionine, and non-significant differences for leucine, isoleucine, arginine, and phenylalanine. Consequently, leucine, isoleucine, and phenylalanine were excluded as contributors to Dbs bitterness. Integrating non-targeted metabolomics data, tryptophan was identified as a potential compound responsible for bitterness in Dbs.

#### 2.3.3. Targeted Metabolomic Analysis of Flavonoids

Targeted metabolomic analysis of flavonoids identified only two differential metabolites: the level of apigenin [40] was significantly higher in Dbs than in Db (*p* < 0.01, Figure 3C), while the concentration of naringenin [41]—a known bitter compound—was lower in Dbs. More importantly, the contents of both bitter compounds in Dbs and Db were far below the human taste detection threshold (TAV << 1, Table 2). Therefore, although they may contribute to the baseline bitterness, their variation in content does not explain the significantly higher bitterness intensity observed in Dbs. Consequently, subsequent investigations prioritized phenolic acids and amino acid derivatives, which showed more pronounced differential accumulation.

### 2.4. Comparative Analysis of Differential Bitter Metabolites Against BitterDB with TAV Assessment

To further identify the potential metabolites responsible for bitterness in Dbs, concentrations of bitter differential metabolites quantified by targeted metabolomics (Table S3) were compared with human bitterness thresholds from the BitterDB database. Taste Activity Values (TAV) were calculated for each compound (Table 2). Results revealed that except for 4-Hydroxybenzoate, none exceeded human bitterness thresholds. Crucially, only 4-Hydroxybenzoate exhibited a TAV > 1 (14.58), while all others showed TAV < 1. Based on threshold comparisons and TAV data, 4-Hydroxybenzoate was established as the sole metabolite significantly contributing to Dbs bitterness perception.

### 2.5. Determination Results of Soluble Sugar Content in Two Bamboo Shoot Varieties

**Figure 4:**
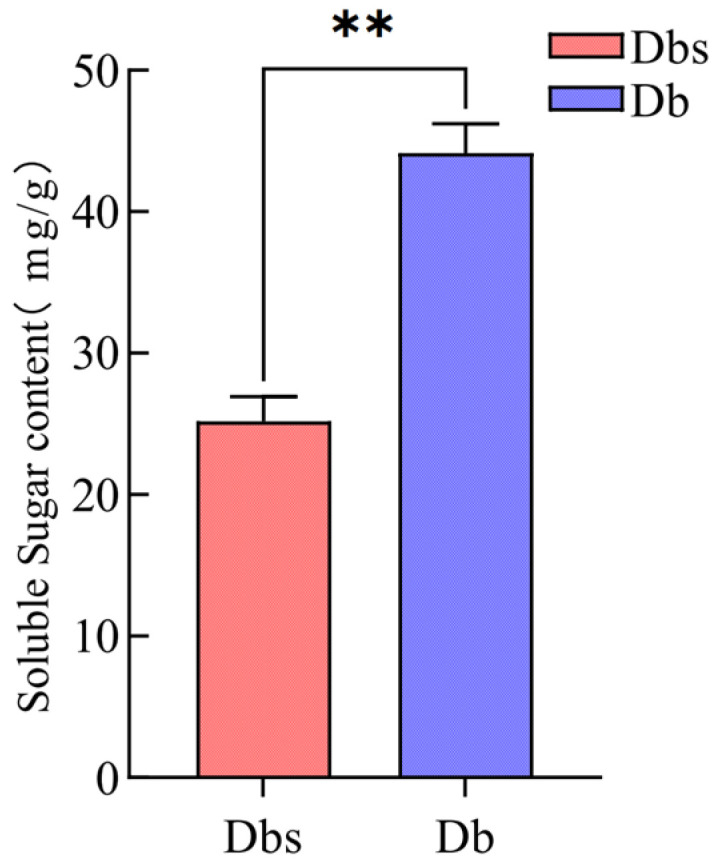
Comparison of Soluble Sugar Contents Between Two Bamboo Shoot Varieties. ** indicates *p* < 0.01.

The soluble sugar content differed significantly between the two varieties, with Db containing markedly higher levels than Dbs (44.143 vs. 25.184 mg/g; *p* < 0.01; Figure 4). Given that sweet and bitter tastes have a competitive inhibition relationship [56,57,58,59], the significantly higher soluble sugar content in Db may contribute to its overall taste profile by counteracting the baseline bitterness detected by the electronic tongue.

### 2.6. Comparative Transcriptomics Analysis of Two Bamboo Shoot Varieties

**Figure 5:**
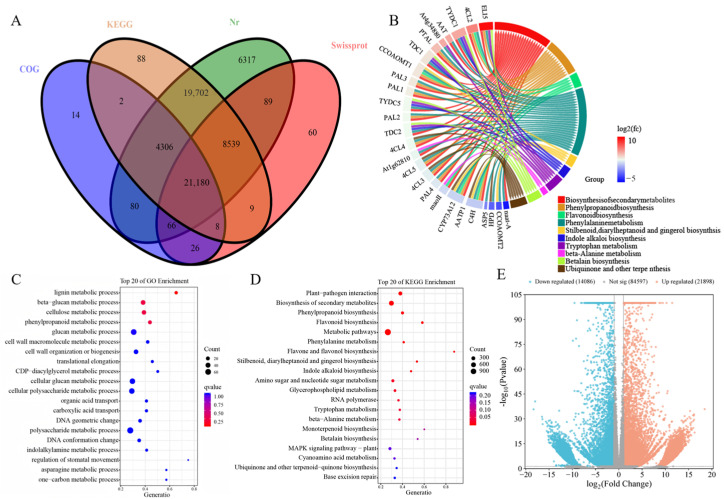
Transcriptomic Analysis Results of “Dbs”: (**A**) Comparison Results of Unigenes against Four Major Databases; (**B**) Volcano Plot of Differentially Expressed Genes (DEGs); (**C**) Top 20 Enriched Pathways in GO Biological Process; (**D**) Top 20 Enriched KEGG Pathways; (**E**) Phenylpropanoid Metabolic Pathway Differential Gene Enrichment Chord Diagram.

Transcriptomic analysis investigating the molecular mechanisms underlying bitterness formation in Dbs yielded 47.69 GB of clean bases after filtering and quality control, with an average Q30 of 93.20% and GC content of 54.87%, identifying 120,582 expressed genes. Unigene sequences aligned against four databases Nr, KEGG, COG, SwissProt; (Figure 5A) showed annotation rates of 60,279 unigenes (50.00% total) for Nr, 53,834 (44.64%) for KEGG, 25,682 (21.30%) for COG, and 29,977 (24.86%) for SwissProt. The PCA results (Figure S1) demonstrated variance explanations of 95.5% (PC1), 2.6% (PC2), and 98.1% cumulative, effectively preserving sample information. Correlation heatmap analysis (Figure S2) confirmed high inter-sample consistency with all biological replicates exhibiting coefficients > 0.9.

Differential expression analysis based on transcriptomic sequencing data identified significantly differentially expressed genes (DEGs) using thresholds of FDR < 0.05 and |log_2_FC| > 1. Compared to Db, Dbs exhibited 30,255 DEGs, comprising 18,520 upregulated and 11,735 downregulated genes (Figure 5E). To elucidate the biological functions of these DEGs, Gene Ontology (GO) and KEGG Orthology (KO) enrichment analyses were performed (Figure S3). GO enrichment revealed 21 significantly enriched biological process terms, 13 cellular component terms, and 11 molecular function terms. Within biological processes, four pathways showed highly significant enrichment: lignin metabolic process, beta-glucan metabolic process, cellulose metabolic process, and phenylpropanoid metabolic process (Figure 5C). KO enrichment identified 131 pathways, with q-value minimization analysis (Figure 5D) demonstrating primary enrichment in Secondary metabolite biosynthesis (622 genes), Plant-pathogen interaction (274 genes), and Phenylpropanoid biosynthesis (114 genes) after excluding the overarching Metabolic pathways.

Significant differential enrichment was observed in phenylpropanoid-related biosynthesis and metabolism across both GO biological processes and KO analyses. Integrated analysis of shared genes from the GO term phenylpropanoid metabolic process and the KO pathway Phenylpropanoid biosynthesis (Figure 5B) revealed predominant enrichment in Biosynthesis of secondary metabolites and Phenylalanine metabolism—the latter being a subset of phenylpropanoid metabolism. Critically, the biosynthesis of 4-Hydroxybenzoate (previously identified as a key bitter compound in Dbs through metabolomics) is mechanistically linked to phenylpropanoid pathways. Consequently, subsequent investigations will prioritize genes within phenylpropanoid metabolism/biosynthesis and reconstruct 4-Hydroxybenzoate synthesis pathways based on KEGG annotations.

### 2.7. Transcriptomic qRT-PCR Validation Results

To further validate the accuracy of transcriptomic sequencing data, primers were designed for significantly differentially expressed genes randomly selected from nine candidate genes, with the stably expressed glyceraldehyde-3-phosphate dehydrogenase gene *GAPDH* serving as the reference gene. qRT-PCR validation demonstrated expression trends consistent with transcriptomic analysis (Figure S4), confirming the reliability of the sequencing results.

### 2.8. Analysis of Key Enzyme Activities in 4-Hydroxybenzoate Biosynthetic Pathway and Corresponding Gene qRT-PCR Validation

**Figure 6:**
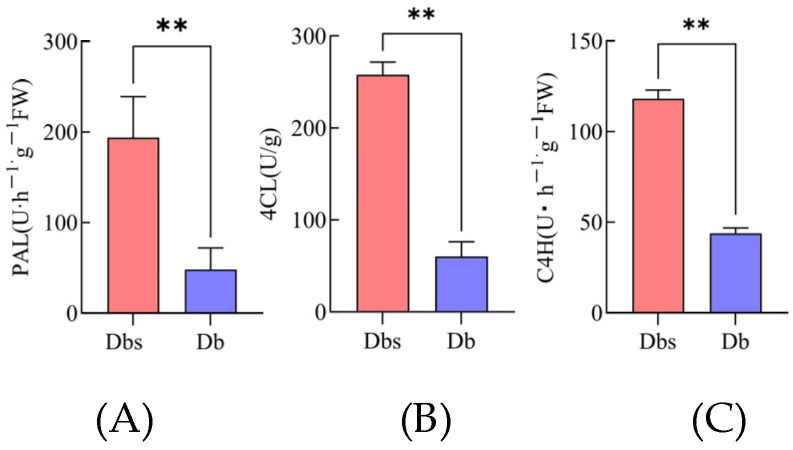
Comparison of Key Enzyme Activities in the Biosynthesis of 4-Hydroxybenzoate Between Two Bamboo Shoot Species. Phenylalanine Ammonia-Lyase, PAL (**A**); 4-Coumarate: Coenzyme A Ligase, 4CL (**B**); Cinnamate 4-Hydroxylase, C4H (**C**). ** indicates *p* < 0.01.

Enzyme activity assays of PAL, C4H, and 4CL enzymes in Db and Dbs, along with qRT-PCR of key genes, identified critical regulators in 4-Hydroxybenzoate biosynthesis. The results (Figure 6A–C) showed significantly higher activities of all three enzymes in Dbs than in Db, with extremely significant differences.

**Figure 7:**
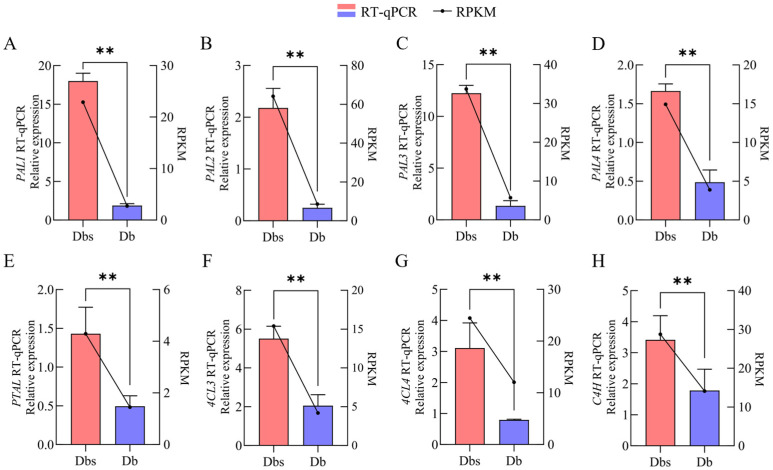
Expression levels of PAL1 (**A**), PAL2 (**B**), PAL3 (**C**), PAL4 (**D**), PTAL (**E**), 4CL3 (**F**), 4CL4 (**G**), and C4H (**H**) genes, along with the validation results of RT-qPCR. The specific RPKM values are provided in Supplementary Table S4. ** indicates *p* < 0.01.

qRT-PCR analysis of key genes *4CL3*, *4CL4*, *PAL1*, *PAL2*, *PAL3*, *PAL4*, *PTAL*, and *C4H* (Figure 7A–H) indicated that the expression levels of these genes in Dbs were significantly higher than those in Db. These results align with transcriptomic data and enzyme activity assays, showing significantly higher expression in Dbs than in Db. This demonstrates that overexpression of these genes in Dbs enhances 4-Hydroxybenzoate synthesis, thereby contributing to bitterness in Dbs.

### 2.9. Analysis of Bitter Compound Biosynthetic Pathway in Specific Germplasm Dendrocalamus brandisii

**Figure 8:**
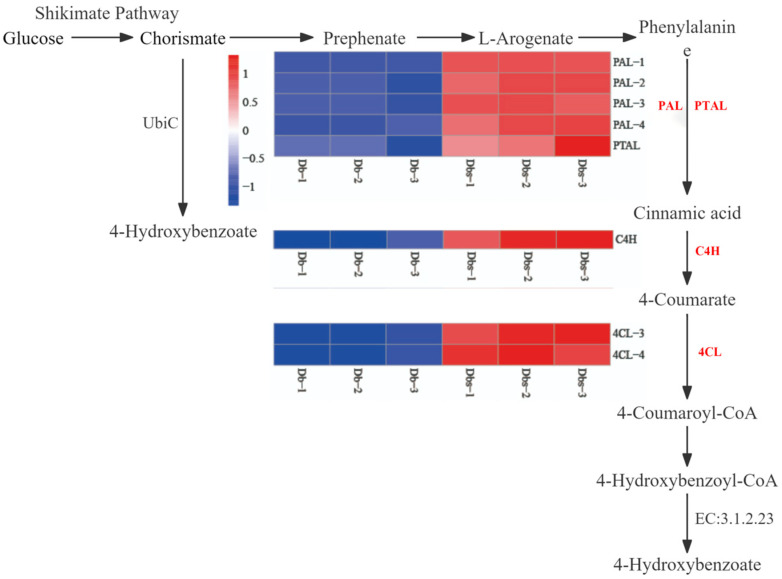
Pathway diagram for synthesis of 4-Hydroxybenzoate in bamboo shoots. The genes highlighted in red are the key genes.

Integrated metabolomic and transcriptomic analyses constructed the biosynthetic pathway of potential bitter compounds in Dbs based on DAMs (Differentially Accumulated Metabolites), DEGs (Differentially Expressed Genes), and KEGG Pathway annotations. Bitter compound synthesis is primarily mediated by enzymes from the phenylpropanoid metabolism pathway (*PAL*, *C4H*, *4CL*, *PTAL*) and the chorismate pathway (Figure 8). Chorismate can be converted to 4-Hydroxybenzoate via chorismate lyase (ubiC, EC:4.1.3.40) or further metabolized to phenylalanine for entry into phenylpropanoid metabolism. Phenylalanine is catalyzed by PAL and PTAL to form cinnamic acid, which enters the ubiquinone pathway. Cinnamic acid is hydroxylated by C4H to yield 4-coumarate. Subsequently, 4-coumarate is activated by 4CL to form 4-coumaroyl-CoA. 4-Coumaroyl-CoA reacts with CoA to produce 4-hydroxybenzoyl-CoA, which is finally hydrolyzed by 4-hydroxybenzoyl-CoA thioesterase (EC: 3.1.2.23) to synthesize 4-Hydroxybenzoate, thereby conferring bitterness to Dbs.

## 3. Discussion

This study revealed that the dramatic accumulation of 4-Hydroxybenzoate in Dbs is the most significant factor contributing to its bitterness, based on its exceptionally high taste activity value (TAV) and upregulation of its biosynthetic pathway. Our data therefore indicate 4-hydroxybenzoate as the predominant bitter compound responsible for the sensory divergence between Dbs and Db. The connection between the chemical profiles and the sensory outcome was established through the integrated analysis of concentration data and taste thresholds. Specifically, the Taste Activity Value (TAV) served as a critical bridge, quantifying how much each compound’s concentration in Dbs exceeded its human bitterness threshold. The singular and decisive case of 4-Hydroxybenzoate—with both a massive concentration fold-change and a TAV far exceeding 1—provides a clear and quantitative link to explain the markedly higher bitterness intensity recorded by the electronic tongue for Dbs compared to Db. As an important plant secondary metabolite, 4-hydroxybenzoate exhibits anti-inflammatory and antioxidant properties [60,61] and originates from the phenylpropanoid metabolic pathway. Numerous intermediates in this pathway are known to impart bitterness, including hydroxycinnamic acid [24,62], L-phenylalanine [24], arbutin [20], phenylpropane glycerol glucoside [63], and amygdalin [64]. Beyond its role in Dbs bitterness identified in this study, 4-hydroxybenzoate has also been associated with bitter tastes in wine fermentation processes [65] and serves as one of multiple phenolic acids contributing to bitterness in pulses [33]. Additionally, 4-hydroxybenzoate has been implicated in autotoxic effects observed in certain plant species [66].

Targeted metabolomic analysis of amino acids in Dbs and Db revealed ten amino acids with higher levels in Dbs than in Db. Among these, glutamic acid, aspartic acid, and glutamine are not bitter amino acids [67]. Gao et al. identified phenylalanine, guanosine, tryptophan, ornithine, and adenine as components contributing to bitterness in bitter bamboo shoots, with phenylalanine being the primary contributor [24]. However, in this study, phenylalanine showed no significant difference between the two bamboo shoot types. Although tryptophan was significantly higher in Dbs than in Db, it did not reach the bitterness threshold [35]. Research by Kirsten Günther-Jordanland et al. on oat bitterness identified leucine, isoleucine, and phenylalanine as contributors to oat bitterness [50]. Although the three bitter amino acids leucine, isoleucine, and phenylalanine are present in both varieties, their concentrations are below the respective human detection thresholds [45,50,51], indicating they are not the primary drivers of perceived bitterness. In contrast, 4-Hydroxybenzoate, while also below the threshold in Db, accumulates to a level 92-fold higher in Dbs, exceeding its threshold and thereby serving as the key compound responsible for the distinct bitter intensity of Dbs. However, the bitter-tasting compounds responsible for the bitterness of bamboo shoots vary across different bamboo shoot species. For instance, integrated metabolomic studies have revealed a diversity of primary bitter compounds across species: flavonoids in *Bambusa rigida* [17]; tannins, flavonoids, and cyanides in *Neosinocalamus affinis* [17]; a correlation with tannins, specific amino acids, and cyanides in *Dendrocalamus latiflorus* [18]; and cyanide accumulation in *Pleioblastus amarus* [19]. The application of integrated transcriptomic and metabolomic analyses has been key to elucidating such bitter taste formation, as demonstrated in *Dendrocalamopsis oldhamii* [20] and *Phyllostachys edulis* [24].

Beyond the spectrum of bitter compounds, we also analyzed differences in soluble sugar content between the two types of bamboo shoots and found that the content in Db was significantly higher than in Dbs. Critically, this chemical difference was directly reflected in human perception, with Db receiving significantly higher sweetness scores (Table 1). In taste perception, sweetness is known to exert a masking effect on bitterness [68,69,70]. Therefore, we propose that the overall sensory difference between Db and Dbs arises from a dual mechanism: (1) the potent and singular accumulation of the bitter agonist 4-Hydroxybenzoate in Dbs, and (2) the potentially stronger inhibitory effect on bitterness in Db due to its richer sugar profile. This integrated perspective shifts the focus from a single bitter compound to the balance between opposing taste stimuli as the key determinant of overall flavor. In this study, untargeted and targeted metabolomics were employed to quantify a spectrum of metabolites. The primary strategy involved screening for bitter compounds by cross-referencing with the BitterDB database. This focused screening, complemented by Taste Activity Value (TAV) calculation, led to the identification and prioritization of 4-Hydroxybenzoate as the key contributor to the intense bitterness in Dbs.

The assessment of bitter taste intensity can be approached through multiple methodologies, each with distinct advantages. Human sensory analysis provides direct perceptual data but can be subject to individual variability. Cellular assays, such as those using heterologously expressed human bitter taste receptors (TAS2Rs), offer mechanistic insights at the molecular level. In this study, we employed an electronic tongue (E-tongue) as a complementary and objective tool for quantitative sensory analysis. The E-tongue provides direct taste readings of sample solutions, including sourness, sweetness, bitterness, astringency, umami, saltiness, bitter aftertaste, astringent aftertaste, and umami richness, enabling quantitative taste analysis through response intensity [71]. This study used an E-tongue to analyze the sensory taste differences between Db and Dbs, finding that the bitterness value of Dbs was significantly higher than that of Db. Currently, E-tongue technology finds extensive application in taste analysis. Chen et al. employed an E-tongue for sensory evaluation of wolfberries from different regions, investigating the correlation with manual sensory evaluation. Their research found good correlation between the sour, salty, umami, sweet, and bitter tastes from manual sensory evaluation and the response values obtained from the E-tongue, confirming that E-tongue technology can serve as an effective substitute for sensory evaluation to objectively and accurately describe the taste characteristics of wolfberries [72]. Zhong et al. used an E-tongue to analyze the sensory characteristics of Huangjin tea, with results consistent with consumer perception [73], further demonstrating that the E-tongue can accurately reflect human taste perception with high sensitivity and objectivity.

In the field of bitter compound research, metabolomic analysis is widely used to screen key metabolites. Huang Shan et al., by combining sensory evaluation and metabolomic analysis to detect changes in bitterness in green prickly ash (*Zanthoxylum schinifolium*) pericarps at different growth stages, successfully identified and screened 18 potential bitter metabolites [74]. Metabolomic comparison between bitter and non-bitter bamboo shoot samples revealed abnormal accumulation of amino acids and phenolic acids in bitter shoots, which was less pronounced in non-bitter shoots. Although the concentrations of these bitter metabolites in the bitter shoots did not reach the bitterness threshold, their accumulation might still contribute to the bitterness of Dbs. Jin Xiaojie et al. conducted metabolomic and transcriptomic analyses on shoot tips of three sweet potato varieties with varying degrees of bitterness and astringency, identifying various flavonoid glycosides and hydroxycinnamic acid derivatives as the main bitter and astringent compounds in sweet potato tips [35]. In this study, hydroxycinnamic acids and flavonoid glycosides showed no difference between the two bamboo shoots. Xiaoxue Li et al. used metabolomics to analyze the cause of bitterness in Chinese winter jujube during ultra-low oxygen storage, finding that the accumulation of coumarin, shikimic acid, and 3-hydroxybenzoic acid during storage led to the development of bitterness [75].

Transcriptome sequencing enables the rapid and precise acquisition of detailed information on the sequences and expression levels of all transcripts in a specific organism under specific conditions [76]. Currently, this technology has become a crucial analytical tool in molecular biology and is widely applied across numerous research fields. Li Qianqian et al. screened key enzymes (SgPAL1-2, SgC4H, Sg4CL, SgTAT1-2, SgHPPR1-2, SgRAS, and SgC3H) in the rosmarinic acid (RA) biosynthesis pathway in Sarcandra glabra based on transcriptomic analysis [77]. It can be observed that the initial part of the rosmarinic acid synthesis pathway, similar to that of 4-Hydroxybenzoate, belongs to the phenylpropanoid pathway. PAL, C4H, and 4CL sequentially catalyze L-phenylalanine (L-phe) into p-coumaroyl-CoA, a key precursor for phenolic acids and flavonoids [31,78,79], which then enters other pathways. Cao Yurong et al. integrated transcriptomics and physiological methods to analyze the reasons for the high cadmium tolerance in *Dendrocalamus brandisii*, discovering that the phenylpropanoid biosynthesis pathway was activated under cadmium stress, leading to increased phenolic compound content and reduced damage [80]. This demonstrates that phenylpropanoid metabolism is not only a key pathway for the formation of bitter compounds in plants but also plays a significant role in plant stress resistance. This study, based on transcriptomic analysis, revealed the relationship between the bitterness of Dbs bamboo shoots and gene expression levels. By comparing gene expression profiles among different samples, differential expression of genes related to phenylpropanoid metabolism—phenylalanine ammonia-lyase [81], 4-coumarate-CoA ligase (4CL), and cinnamate 4-hydroxylase (C4H)—was identified in bitter shoots. These differentially expressed genes may be associated with the biosynthesis of the bitter compound 4-Hydroxybenzoate. Analysis of the enzymatic activity of key enzymes (PAL, 4CL, C4H) in the 4-Hydroxybenzoate synthesis pathway showed significantly higher activity in Dbs than in Db, consistent with the transcriptomic results. This further indicates that the accumulation of 4-Hydroxybenzoate is the cause of bitterness in Dbs.

This study identified 4-Hydroxybenzoate as the key bitter compound in Dbs. However, a subsequent question emerges: Why do plants need to synthesize this compound? Research has revealed that 4-Hydroxybenzoate is explicitly recognized as an allelochemical in plants, which can be released into the soil via root systems to inhibit seed germination and root growth of the plant itself or neighboring plants [82]. For example, it acts as an autotoxin in cucumber root exudates, suppressing root growth by reducing the activity and length of meristematic cells [83], and this property may play a crucial role in continuous cropping obstacles [84]. Additionally, 4-Hydroxybenzoate is involved in plant stress defense and structural regulation. In carrots, 4-Hydroxybenzoate can covalently bind to newly synthesized cell wall polysaccharides, enhancing the impermeability and mechanical strength of the cell wall, thereby improving the plant’s tolerance to freezing stress or pathogen infection [81]. In poplars, 4-Hydroxybenzoate mediates the specific modification of lignin via the PHBMT1 enzyme; this modification is induced when plants respond to gravity or mechanical stress, and then negatively regulates the plant’s gravitropic response, maintaining the growth posture and structural balance of trees [85]. Therefore, the abnormally high accumulation of 4-Hydroxybenzoate in Dbs may not only be a manifestation of its bitter trait but also a defensive state of this special germplasm under specific contexts or a continuous stress response to its growth microenvironment. In this study, the general upregulation of key genes in the phenylpropanoid metabolic pathway and the enhancement of corresponding enzyme activities in Dbs lead us to speculate that this may be due to natural genetic variations in the Dbs germplasm (such as promoter variations or copy number variations in key transcription factors), resulting in its defense system being in an “alert” state. Although this state manifests as an undesirable bitter taste in human taste perception, for the plant itself, it may be an adaptive mechanism to improve stress resistance.

In conclusion, this study identified 4-Hydroxybenzoate as the key bitter component responsible for the intensified bitterness in Dbs shoots and elucidated its biosynthetic pathway. It is important to note that our assessment was based on the contribution of individual compounds. To fully decipher the complex bitterness of Dbs, future research should not only further explore the gene regulatory networks and environmental impacts on bitter compound accumulation but also investigate potential synergistic effects among multiple taste-active compounds, which were beyond the scope of the present targeted analysis. Approaches such as sensory recombination experiments will be crucial to understand these interactions. Furthermore, the electronic tongue analysis in this study provided a robust comparative assessment of relative bitterness between Db and Dbs. For future investigations aiming at absolute quantification of bitterness intensity, the inclusion of a standard bitter compound as a positive control would be advantageous to calibrate the sensor response and facilitate cross-study comparisons. Such integrated knowledge will provide a comprehensive foundation for improving the taste and edible quality of bamboo shoots.

## 4. Materials and Methods

### 4.1. Materials

The bamboo shoots of *D. brandisii* (Db) were collected from the bamboo forest in Banpo Village, Yixiang Township, Simao District, Pu’er City, Yunnan Province, China (22.75° N, 101.13° E). The bamboo shoots of *D. brandisii* of specific germplasm (Dbs) were collected from the cultivated clump at Southwest Forestry University, Yunnan Province, China (N 25°06′, E 102°78′). This clump of bamboo was transplanted from a local nursery in Honghe Prefecture, Yunnan Province to the university campus in 2014. After several years of propagation, the morphological characteristics of these bamboo plants and their shoots are identical to those of the common *D. brandisii* (Db). However, they differ significantly in taste: Db exhibits a sweet flavor, whereas Dbs displays a pronounced bitter taste. The shoots of both Db and Dbs were harvested at a comparable growth stage. They were approximately 30–32 cm in length and 10–12 cm in basal diameter. The entire edible portion of each shoot was used for subsequent analyses to ensure a representative sample. All shoots exhibited vigorous growth, were free from pests and diseases, and showed no significant external morphological differences. The sampling work was carried out in mid-August 2022. Three biological replicates, each comprising multiple shoots, were collected per bamboo shoot type. Freshly harvested shoots were immediately processed. For electronic tongue analysis, the shoots were first peeled and rinsed with pure water, they were then juiced using a juicing extractor (Enerburg ALW-J19). To prevent oxidative degradation that could alter taste, 0.25 mL of a 1% (*w*/*v*) ascorbic acid solution was added per 50 mL of raw juice immediately after extraction. The juice was filtered through three layers of non-woven fabric. Subsequently, 50 mL aliquots of the filtrate were transferred into pre-cooled amber glass bottles (prepared in triplicate per treatment) and subjected to electronic tongue analysis. Another portion was rapidly minced into fragments 3 cm × 1 cm × 1 cm (L × W × H) on a liquid-nitrogen-cooled surface using a sterile ceramic knife, immediately transferred into sterile RNase-free 50 mL tubes (three biological replicates per treatment), flash-frozen in liquid nitrogen, and stored at −80 °C until analysis to prevent degradation.

### 4.2. Electronic Tongue Analysis and Sensory Evaluation of Two Bamboo Shoot Varieties

#### 4.2.1. Analysis of Bitterness Intensity in Bamboo Shoots from the Specific Germplasm of *Dendrocalamus brandisii*

The 50 mL filtered bamboo shoot solutions of Dbs and Db were sent to the Kunming Sci-go Instrument Testing Platform for analysis using a Japanese Insent SA402B electronic tongue system. The electronic tongue bitterness analysis system comprises six sensors (C00, AE1, CA0, CT0, AAE, GL1) and three reference electrodes (Table 3).

Taste analysis was performed using an electronic tongue (SA402B model, Insent, Atsugi, Japan). The cleaning and reference solutions were prepared as follows: the anode cleaning solution contained 7.46 g of KCl, 0.56 g of KOH, 300 mL anhydrous ethanol, and was diluted to 1000 mL with purified water; the cathode cleaning solution contained 8.3 mL concentrated HCl (37%, *w*/*w*), 300 mL anhydrous ethanol, and was diluted to 1000 mL with purified water; the reference solution contained 2.24 g of KCl and 0.045 g of tartaric acid, diluted to 1000 mL with purified water. Following the method described by Ruixin Liu [86], sample solutions were centrifuged at 3000 rpm for 3 min, and 35 mL of supernatant was aliquoted for measurement. Prior to analysis, the sensors were cleaned in the cleaning solutions for 90 s and equilibrated in the reference solution for 120 s. After a 30 s zeroing operation, taste detection was conducted for 30 s. For aftertaste measurement, sensors were rinsed in reference solution for 3 s before a 30 s immersion in fresh reference solution. This cycle was repeated five times, with data from the middle three cycles averaged as final results. The tasteless point for sourness was set to −13 due to the tartaric acid in the reference solution, while for other tastes it was set to zero.

Following data collation and summarization, biostatistical analysis (Mean ± SD) was performed using independent samples *t*-test in SPSS Statistics 27.0 (R27.0.1.0) software, and results were visualized using GraphPad Prism 10.1.2 software.

#### 4.2.2. Human Sensory Evaluation

A total of 12 sensory assessors (aged 23–25 years) were recruited for sensory evaluation, with screening and training conducted via the three-point test in compliance with the international standard ISO 4120:2021. First, assessors performed taste identification on aqueous solutions of quinine hydrochloride [45,87] (0.0025 g/L) and sucrose [88,89] (2 g/L) at their respective basic taste threshold concentrations. Second, for each of the two tastes, six dilution gradients were prepared in ascending order of mass concentration—each system comprising “pure water (0 points, tasteless)” and five concentration-increasing gradients. These gradients were provided to assessors for identifying the same taste at varying intensities, alongside systematic training on the taste intensity scale. Ultimately, 10 qualified assessors were selected. Samples were uniformly processed, cut into 1 cm × 1 cm × 2 cm pieces, and served in coded white paper cups at room temperature. During formal evaluation, each assessor received three samples (two identical, one distinct) under a randomized balanced design: they first completed the three-point test to identify the odd sample, then rated the sweetness and bitterness intensities (0–5 scale) of the two target samples. Paired-samples *t*-tests were applied to the collected data to compare the sweetness and bitterness intensity scores between the two samples, thereby quantifying their flavor profiles. The training tables for sweetness and bitterness, experimental master control table, and questionnaire are presented in Table S7.

### 4.3. Non-Targeted Metabolomic Analysis of Both Bamboo Shoot Types

Frozen samples stored at −80 °C were first lyophilized. The freeze-dried samples were crushed using a mixer mill (MM 400, Retsch, Haan, Germany) with a zirconia bead for 1.5 min at 30 Hz. 100 mg of the resulting powder was added to 1.0 mL of 70% aqueous methanol containing 0.1 mg/L internal standard (lidocaine) for overnight extraction, with three vortex-mixing steps during extraction to enhance efficiency. After extraction, samples were centrifuged at 10,000 g for 10 min. The supernatant was filtered through a 0.22 μm microporous membrane and analyzed using an LC-ESI-MS/MS system. The chromatographic separation was carried out on a Waters ACQUITY UPLC HSS T3 C18 column (1.8 µm, 2.1 × 100 mm) using a mobile phase of (A) water with 0.04% acetic acid and (B) acetonitrile with 0.04% acetic acid at a flow rate of 0.4 mL/min. The gradient elution program was: 0 min, 95% A; 11.0 min, 5% A; hold until 12.0 min; return to 95% A at 12.1 min and hold until 15.0 min. The column temperature was maintained at 40 °C and the injection volume was 2 µL. The analysis was performed on the aforementioned system (UPLC: Shim-pack UFLC SHIMADZU CBM30A (Kyoto, Japan); MS/MS: Applied Biosystems 6500 QTRAP (Waltham, MA, USA)) for detection and data acquisition. Quality control was ensured using internal standards and metabolite QC samples. Metabolites were quantified in Multiple Reaction Monitoring (MRM) mode with a predefined MRM method [90]. Acquired mass spectrometry data were processed and analyzed using Analyst 1.6.1 software (AB Sciex, Framingham, MA, USA). Differential metabolites between Db and Dbs were screened based on VIP ≥ 1 and *t*-test *p* < 0.05. Significantly differential metabolites were visualized using TBtools (v1.098684) and subjected to KEGG path-way analysis.

### 4.4. Targeted Metabolomic Analysis of Both Bamboo Shoot Types

High-throughput targeted quantification of phenolic acids, amino acids, and flavonoids was performed for both bamboo shoot types. Based on the detection data, quantitative analysis of the samples was conducted. Statistical analysis (mean ± SD) was performed using independent samples *t*-test in SPSS Statistics 27.0 software. Finally, results were visualized using GraphPad Prism 10.1.2 software.

#### 4.4.1. Extraction and Detection of Phenolic Acid Metabolites

Precisely weighed standard compounds (for comprehensive details regarding the standard compounds, please consult Supplementary Table S6) were dissolved in methanol to prepare individual stock standard solutions at 10 mg/mL. An appropriate volume of each stock solution was mixed to prepare mixed standard solutions at 1 μg/mL or 10 μg/mL. Serial dilutions were further prepared to meet the multi-concentration requirements for mass spectrometry analysis. After establishing calibration curves, 0.5 g of frozen plant material was treated with 2 mL of 4 M aqueous NaOH and hydrolyzed at 40 °C for 2 h. The pH was adjusted to 2 using 4 M HCl. The mixture was shaken with 2 mL n-hexane for 20 min at room temperature, and the hexane layer was discarded. The aqueous layer was extracted with ethyl acetate (2 × 2 mL). Combined extracts were concentrated to near-dryness under reduced pressure at 35 °C using a rotary evaporator. Residues were reconstituted in 200 μL of methanol:water (1:1, *v*/*v*) prior to analysis. Samples were centrifuged at 12,000 rpm for 10 min. The supernatant was subjected to LC (Vanquish, UPLC, Thermo, Waltham, MA, USA)-MS (Q Exactive, Thermo, Waltham, MA, USA) analysis for data acquisition. The liquid chromatography separation was performed on a Waters HSS T3 column (50 × 2.1 mm, 1.8 μm) using a mobile phase consisting of (A) ultrapure water with 0.1% acetic acid and (B) acetonitrile with 0.1% acetic acid, at a flow rate of 0.3 mL/min. The column temperature was maintained at 40 °C, and the injection volume was 2 μL. The gradient elution program was set as follows: 0–2 min, 90% A; 2–6 min, A decreased linearly from 90% to 40%; 6–8 min, maintained at 40% A; 8.1–12 min, returned to 90% A for equilibration. Samples were kept at 4 °C in the autosampler and analyzed in random order, with quality control (QC) samples interspersed to monitor system stability. Mass spectrometry detection was carried out on a Thermo QExactive system equipped with an electrospray ionization (ESI) source operating in negative ion mode. The ESI conditions were: spray voltage, −2800 V; ion transfer tube temperature, 320 °C; sheath gas flow, 40 arb; auxiliary gas flow, 10 arb. The scan range was m/z 100–900, and data were acquired in selected ion monitoring (SIM) mode.

#### 4.4.2. Extraction and Detection of Amino Acid Metabolites

Accurately weighed amino acid standards compounds (for comprehensive details regarding the standard compounds, please consult Supplementary Table S6) were dissolved in methanol or water to prepare individual stock solutions. Appropriate volumes of these stocks were combined to create mixed standard solutions, which were diluted with methanol/water (1:1, *v*/*v*) containing 10% formic acid to achieve working concentrations. Isotope-labeled standards (e.g., Trp-d_3_) were precisely weighed and prepared as 1000 ng/mL internal standard (IS) stock solutions in methanol/water (1:1, *v*/*v*) with 10% formic acid. For sample processing, precisely weighed samples were placed in 2 mL centrifuge tubes. Then, 600 μL of extraction solvent (methanol/water, 1:1, *v*/*v*, with 10% formic acid) was added along with two stainless steel beads. Samples were vortex-mixed for 30 s, processed in a high-throughput tissue homogenizer (MEIBI, MB-96S, Guanajuato, Mexico) at 55 Hz for 90 s, and centrifuged at 12,000 rpm (4 °C) for 5 min. A 100 μL aliquot of the supernatant was diluted 10-fold with 900 μL of methanol/water (1:1, *v*/*v*) containing 10% formic acid, followed by vortex-mixing for 30 s. Subsequently, 100 μL of the diluted supernatant was mixed with 100 μL of 100 ng/mL Trp-d_3_ IS solution and vortexed for 30 s. The mixture was filtered through a 0.22 μm membrane prior to LC-MS analysis for data acquisition.

#### 4.4.3. Extraction and Detection of Flavonoid Metabolites

Accurately weighed flavonoid standard compounds (for comprehensive details regarding the standard compounds, please consult Supplementary Table S6) were dissolved in 80% methanol to prepare stock solutions. Precisely weighed samples were placed in 2 mL centrifuge tubes, mixed with 600 μL methanol, and vortexed for 60 s. Then, 100 mg glass beads were added, and samples were high-throughput tissue homogenizer (MEIBI, MB-96S) at 60 Hz for 1 min using a tissue homogenizer. This homogenization step was repeated at least twice. Subsequently, samples were sonicated at room temperature for 15 min, centrifuged at 12,000 rpm (4 °C) for 5 min, and the supernatant was filtered through a 0.22 μm membrane. The filtrate was subjected to LC-MS analysis for data acquisition.

### 4.5. Screening of Bitter Compounds via Metabolomics and Calculation of TAV

Concentrations of significantly differential bitter compounds in both bamboo shoot types were compared against human bitterness recognition thresholds documented in BitterDB and related studies. For apigenin (lacking a defined threshold), comparisons were made with the minimum effective concentration reported to activate human bitter taste receptors. This identified potential bitter substances responsible for Dbs bitterness. Taste Activity Value (TAV) was calculated as the ratio of taste compound concentration (C) to its taste threshold (T) [91]: TAV = C/T, This metric quantifies the contribution of specific taste components to overall flavor perception [92]. Higher TAVs indicate greater contribution to taste. When TAV > 1, the compound significantly contributes to the sample’s taste profile; when TAV < 1, its contribution is negligible.

### 4.6. Determination Method for Soluble Sugar Content in Bamboo Shoots

The soluble sugar content in bamboo shoots was determined using the anthrone-sulfuric acid colorimetric method. Briefly, approximately 0.10 g of fresh tissue (accurately weighed) was homogenized and extracted twice with 8 mL of distilled water in a boiling water bath for 30 min each. The combined extracts were filtered and diluted to a final volume of 25 mL with distilled water. For color development, a 0.5 mL aliquot of the diluted extract was mixed with 1.5 mL of distilled water, followed by the sequential addition of 0.5 mL of freshly prepared anthrone-ethyl acetate reagent (1% *w*/*v*, prepared by dissolving 1.0 g of anthrone in 50 mL of ethyl acetate) and 5 mL of concentrated sulfuric acid (Caution: added slowly with shaking). The mixture was vortexed immediately and then heated in a boiling water bath for exactly 1 min. After cooling to room temperature, the absorbance was measured at 630 nm. A standard curve was prepared using glucose solutions (0–100 μg) processed identically. The soluble sugar content was expressed as milligrams of glucose equivalents per gram of fresh weight (mg/g FW) based on the standard curve.

### 4.7. Transcriptome Sequencing and Analysis of Both Bamboo Shoot Types

Transcriptome sequencing of bamboo shoot samples (Dbs and Db) was performed by Guangzhou Genedenovo Biotechnology Co., Ltd. (Guangzhou, China) on the Illumina HiSeqTM 4000 platform. After quality control (removing adapter-containing reads, reads with >10% N bases, poly-A reads, and low-quality reads), clean reads were assembled using Trinity software to generate contigs. The resulting unigenes underwent functional annotation to reveal biological characteristics and potential functions. Gene expression levels were quantified as Reads Per Kilobase per Million mapped reads (RPKM) using RSEM software. For subsequent analyses, RPKM values were adopted as the standard metric for gene expression quantification. The expression level of gene A is defined as RPKM(A), where

$$\mathrm{RPKM}={\;10}^{6}C∕\mathrm{NL}∕10^{3}$$

*C*: Number of reads uniquely aligned to gene A; *N*: Total number of uniquely aligned reads in the sample; *L*: Length of gene A in base pairs.

Outlier samples were excluded through PCA analysis and calculation of Pearson correlation coefficients between samples. Differentially expressed genes (DEGs) were identified using DESeq2 with thresholds set at |log2 (fold change)| > 1 and FDR < 0.05. GO enrichment analysis of DEGs was performed using R software (r: the r project for statistical computing) with a significance threshold of *p* < 0.05.

### 4.8. Total RNA Extraction, Transcriptome Validation by qRT-PCR, and Analysis

Total RNA was extracted from *D. brandisii* shoots using the TRIzol method. Reverse transcription was performed using TransScript^®^ All-in-One First-Strand cDNA Synthesis Super Mix for qPCR (One-Stepg DNA Removal) kit (Cat: Q11122) from Beijing TransGen Biotech Co., Ltd. (Beijing, China). Primers for nine randomly selected key genes were designed with Primer 5 based on transcriptome results (Table S1) for validation via quantitative real-time PCR (qRT-PCR). PCR amplification was conducted on a CFX96 Touch Real-Time PCR Detection System (Bio-Rad, Hercules, CA, USA) under the following program: 95 °C for 30 s (initial denaturation), followed by 40 cycles of 95 °C for 5 s (denaturation) and 60 °C for 30 s (annealing/extension). *GAPDH* served as the internal reference gene. Relative gene expression levels were calculated using the 2^−ΔΔCT^ method. Data were processed in Excel and visualized using Origin 2024 (10.1.0.178) software.

### 4.9. Key Enzyme Activity Analysis in the Biosynthesis Pathway of 4-Hydroxybenzoate and qRT-PCR Validation of Related Genes

#### 4.9.1. PAL Enzyme Activity Assay

Frozen samples (3 g) were ground in 9 mL of borate buffer (pH 8.8, containing 1 g PVPP, 5 mM β-mercaptoethanol, and 22 mM EDTA) to obtain a homogenate, which was then centrifuged at 14,000× *g* and 4 °C for 15 min. A 300 μL aliquot of the supernatant was transferred and mixed with 300 μL of boric acid buffer (pH 8.0, containing 20 mM L-phenylalanine) and 600 μL of distilled water. For the control group, the boric acid buffer was replaced with 900 μL of distilled water. The reaction was carried out at 25 °C for 90 min and then deactivated. Following deactivation, the absorbance of trans-cinnamic acid was measured at 290 nm. Enzyme activity was defined as one unit (U) for a change in absorbance of 0.01 per hour, and was expressed as U·h^−1^·g^−1^ fresh weight (FW). All samples were measured in triplicate.

#### 4.9.2. C4H Enzyme Activity Assay

The C4H enzyme activity assay was performed with reference to the method of [58] with modifications. Briefly, 3 g of bamboo shoots were homogenized with 5 mL of extraction buffer (containing 50 mM Tris-HCl buffer pH 8.9, 15 mM β-mercaptoethanol, 4 mM MgCl_2_, 5 mM ascorbic acid, 10 μM leupeptin, 1 mM PMSF, 0.15% (*w*/*v*) PVP, and 10% glycerol) in an ice bath. The homogenate was centrifuged at 12,000× *g* for 20 min at 4 °C. Then, 0.8 mL of the supernatant was mixed with 2.2 mL of reaction buffer (containing 2 μM trans-cinnamic acid, 50 mM Tris-HCl buffer pH 8.9, 2 μM NADPNa_2_, and 5 mM glucose-6-phosphate sodium). Absorbance was immediately measured at 340 nm. The control group used 0.8 mL of distilled water instead of the supernatant. One unit of enzyme activity (U) was defined as an increase of 0.01 in absorbance per hour. Enzyme activity was expressed as U·h^−1^·g^−1^ FW (fresh weight), with three replicates per sample.

#### 4.9.3. 4CL Enzyme Activity Assay

4CL enzyme activity was assayed using a commercial kit. Tissue samples (0.1 g) were homogenized in 1 mL of extraction buffer according to a tissue mass (g) to buffer volume (mL) ratio of 1:10. The homogenate was centrifuged at 8000× *g* and 4 °C for 10 min, and the supernatant was kept on ice for assay. A UV-spectrophotometer was preheated for 30 min and calibrated with distilled water at 333 nm. Following the kit protocol, assay components were added to centrifuge tubes. After thorough mixing with timing initiated immediately, the initial absorbance at 333 nm was recorded as A1_sample_ and A1_blank_. Following a 1 h reaction at 37 °C, the absorbance at 333 nm was measured again and recorded as A2_sample_ and A2_blank_. ΔA_sample_ = A2_sample_ − A1_sample_, ΔA_blank_ = A2_blank_ − A1_blank_, and ΔA = ΔA_sample_ − ΔA_blank_. One unit of enzyme activity was defined as the

$$4\mathrm{CL}\;(U∕g)=(\Delta A\times V_{\mathrm{RS}}\times V_{\mathrm{CT}}\times 10^{9})∕(\varepsilon \;\times \;d\;\times \;W\;\times {\;V}_{\mathrm{RSE}}\;\times \;T)=417.19\;\times \;\Delta A∕W$$

Annotations: V_RSE_: Volume of crude enzyme extract added to the reaction system, 0.1 mL; V_CT_: Total volume of crude enzyme extract, 1 mL; V_RS_: Total volume of the reaction system, 1 × 10^−3^ L; ε: Molar extinction coefficient of 4-coumaroyl-CoA, 2.1 × 104 L/mol/cm; d: Light path length of the quartz cuvette, 1 cm; T: Reaction time, 1 h; W: Sample mass, g.

#### 4.9.4. Validation of Rate-Limiting Enzyme Genes by qRT-PCR

Total RNA was extracted from bamboo shoots using the Trizol method. RNA integrity was assessed by electrophoresis on a 1% agarose gel (120 V, 110 mA) and visualized using a gel imaging system. RNA concentration and purity were determined by measuring the A260/A280 ratio using a NanoDrop2000 spectrophotometer. RNA samples with A260/A280 ratios between 1.8 and 2.0 and concentrations of approximately 500 ng/μL were reverse-transcribed using the TransScript^®^ All-in-One First-Strand cDNA Synthesis Super Mix for qPCR (One-Step gDNA Removal; TransGen Biotech, Cat Q11122). cDNA was subsequently subjected to quantitative PCR with the PerfectStart^®^ Green qPCR SuperMix (TransGen Biotech, Cat AQ601) to validate rate-limiting enzyme genes (primer sequences listed in Table S2).

## 5. Conclusions

This study, by integrating e-tongue sensory evaluation with targeted metabolomic and transcriptomic analyses, determined through Taste Activity Value (TAV) assessment that the drastically elevated accumulation of 4-Hydroxybenzoate (92-fold higher in Dbs than in Db) conclusively explains the significantly heightened bitterness intensity uniquely characteristic of Dbs shoots. This sensory contrast is further modulated by the concurrent, significantly higher soluble sugar content in Db, which was sensorially verified and likely attenuates its baseline bitterness through sweetness-induced masking. The expression levels of key enzymes (PAL, 4CL, C4H) and key genes (*4CL3*, *4CL4*, *PAL1*, *PAL2*, *PAL3*, *PAL4*, and *PTAL*) were all significantly higher in Dbs than in Db. Furthermore, based on KEGG pathway and differentially expressed genes (DEGs) analysis, this study constructed the expression pathway of bitterness-related genes in Dbs and the biosynthetic pathway of key bitter components in bamboo shoots. The expression of these key genes promotes the dynamic accumulation of the bitter metabolite 4-Hydroxybenzoate, resulting in the bitterness of Dbs. These findings provide valuable information for understanding the underlying mechanisms of bamboo shoot bitterness from chemical composition to human perception and promoting the cultivation of high-quality bamboo shoots. Therefore, these results can be utilized to reduce the accumulation of bitter compounds in bamboo, enhancing its edible and economic value, thereby addressing the bitterness issue in Dbs. In summary, this study identified the key bitter compound causing bitterness in bamboo shoots and elucidated its biosynthetic pathway, which will facilitate the development of cultivation and processing strategies to improve the edibility and economic value of bitter bamboo shoots.

## Figures and Tables

**Table 1:** Sensory Evaluation Score Table for Bitterness and Sweetness of Two Bamboo Shoot Varieties.

	Sensory Evaluation Score	
Sample	Sweetness	Bitterness
Dbs	0.6 ± 0.516 a	3.9 ± 0.739 b
Db	3.8 ± 0.632 b	0.7 ± 0.483 a

Note: Different lowercase letters within the same column indicate a significant difference (*p* < 0.01, paired-sample *t*-test).

**Table 2:** Comparison of Taste Activity Values (TAV) of Bitter Compounds in “Dbs” and “Db”.

Substance	Formula	Human Threshold (mM)	Unit Conversion (ng/mg)	Matrix	Concentration in Dbs (ng/mg)	Concentration in Db (ng/mg)	TAV (Dbs)	TAV (Db)
Gallic acid [42]	C_7_H_6_O_5_	<0.001 [32,43]	0.034	Water	0.016	0.008	0.471	0.235
Protocatechuic acid [44]	C_7_H_6_O_4_	0.020 [45]	2.001	Water	0.042	0.032	0.021	0.016
4-Hydroxybenzoate [33]	C_7_H_6_O_3_	0.015 [45]	2.003	Water	29.205	0.314	14.581 *	0.157
Catechin [40]	C_15_H_14_O_6_	0.410 [32,45]	119	Water	0.001	Not detected	<0.001	<0.001
Vanillic acid [46]	C_8_H_8_O_4_	0.060 [45]	9.996	Water	0.256	0.245	0.026	0.025
Epicatechin [40,47]	C_15_H_14_O_6_	0.540 [32]	156.75	Water	0.001	<0.001	<0.001	<0.001
Vanillin [48,49]	C_8_H_8_O_3_	0.300	45.645	Assay Buffer	1.024	0.412	0.022	0.009
Tryptophan [35,50]	C_11_H_12_N_2_O_2_	5.000 [51]	1021.15	Water	58.872	17.314	0.058	0.017
Histidine [34]	C_6_H_9_N_3_O_2_	45.000 [50]	6982.2	Water	77.230	60.774	0.011	0.009
Valine [36]	C_5_H_11_NO_2_	21.000 [52]	2460.15	Water	58.393	39.623	0.024	0.016
Methionine [37]	C_5_H_11_NO_2_S	5.000 [51]	746.05	Water	31.897	24.681	0.043	0.033
Isoleucine [38]	C_6_H_13_NO_2_	10.000 [50]	1311.8	Water	22.515	19.508	0.017	0.015
Leucine [39]	C_6_H_13_NO_2_	11.000 [45]	1442.914	Water	39.917	33.684	0.028	0.023
Phenylalanine [39]	C_9_H_11_NO_2_	45.000 [51]	7433.55	Water	31.945	27.277	0.004	0.004
Naringenin [41]	C_15_H_12_O_5_	0.033 [53,54]	8.984	Water	0.002	0.003	<0.001	<0.001
Apigenin [55]	C_15_H_10_O_5_	0.001 [40]	0.270	Dimethyl Sulfoxide	0.014	0.008	0.052	0.030

* indicates key bitter compounds.

**Table 3:** Matching information between sensor and taste value.

Sensor	The Corresponding Taste	Taste Information	
		Taste First	Aftertaste
C00	Acidic bitterness	Bitterness	Aftertaste-B
AE1	Astringency	Astringency	Aftertaste-A
CA0	Sourness	Sourness	×
CT0	Saltness	Saltness	×
AAE	Umami	Umami	Richness
GL1	Sweetness	Sweetness	×

Note: “×” denotes the absence of aftertaste.

## Data Availability

Data will be made available on request.

## Supplementary Materials

The following supporting information can be downloaded at: https://www.mdpi.com/article/10.3390/plants15040560/s1, Figure S1: Principal Component Analysis (PCA) results of the samples. Figure S2: Sample correlation heat map. Figure S3: GO enrichment analysis results. Figure S4: Validation results of transcriptome sequencing by qRT-PCR; Table S1: Primer sequence list. Table S2: Primer synthesis sequence list. Table S3: Non-targeted metabolomics screening for metabolites associated with differences in bitterness. Table S4: Transcripts of key genes. Table5: Detailed information of non-targeted metabolomic compounds. Table S6: Information on targeted metabolic standards for phenolic acids, amino acids, and flavonoids. Table S7: Master Control Sheet, Evaluation Form, and Questionnaire for Sensory Evaluation.
