# Supplementary material for: Integrated Metabolomics and Transcriptomics Reveal Bitter Compounds and Synthetic Pathways in the Special-Germplasm Bitter-Tasting Dendrocalamus brandisii
Source: Plants (Basel). 2026 Feb 10;15(4):560. doi: 10.3390/plants15040560 (PMC12943958; doi:10.3390/plants15040560)
Supplement: Supplementary file 1 [file plants-15-00560-s001.zip › Supplementary Figure.pdf]

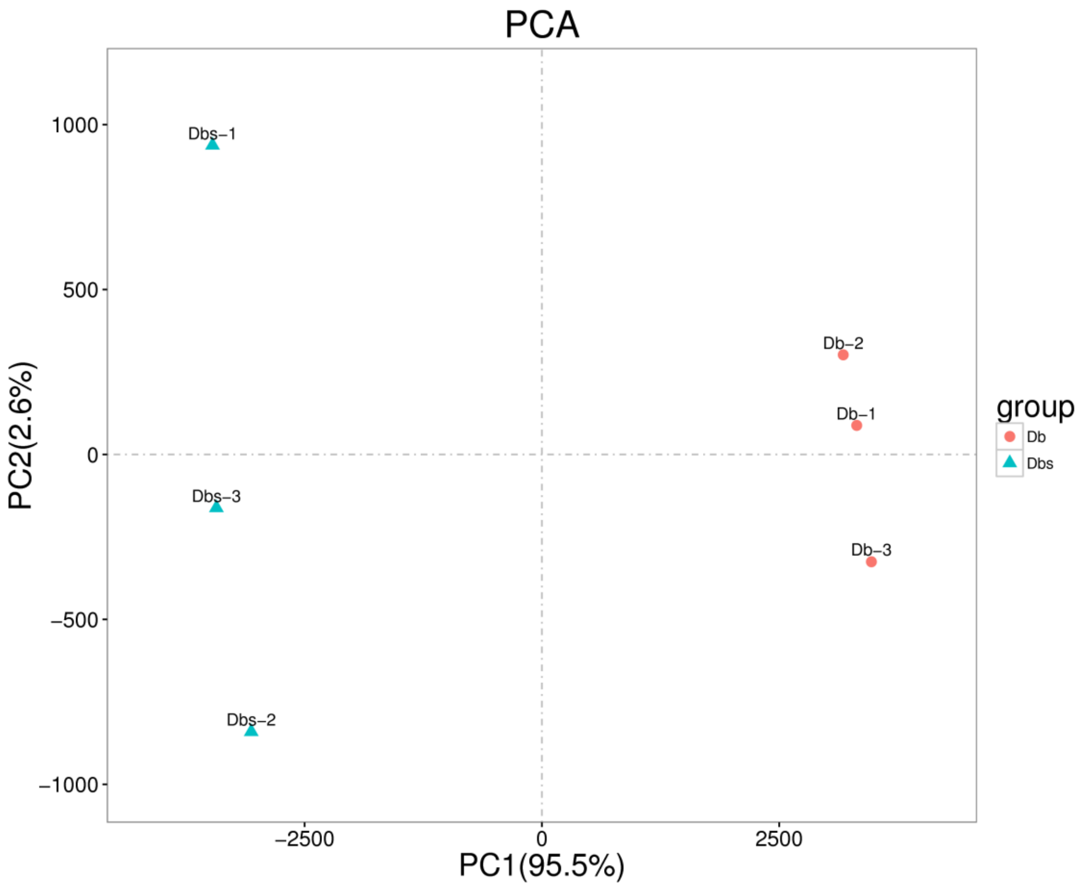

Fig.S1 PrincipalComponentAnalysis(PCA)resultsofthesamples

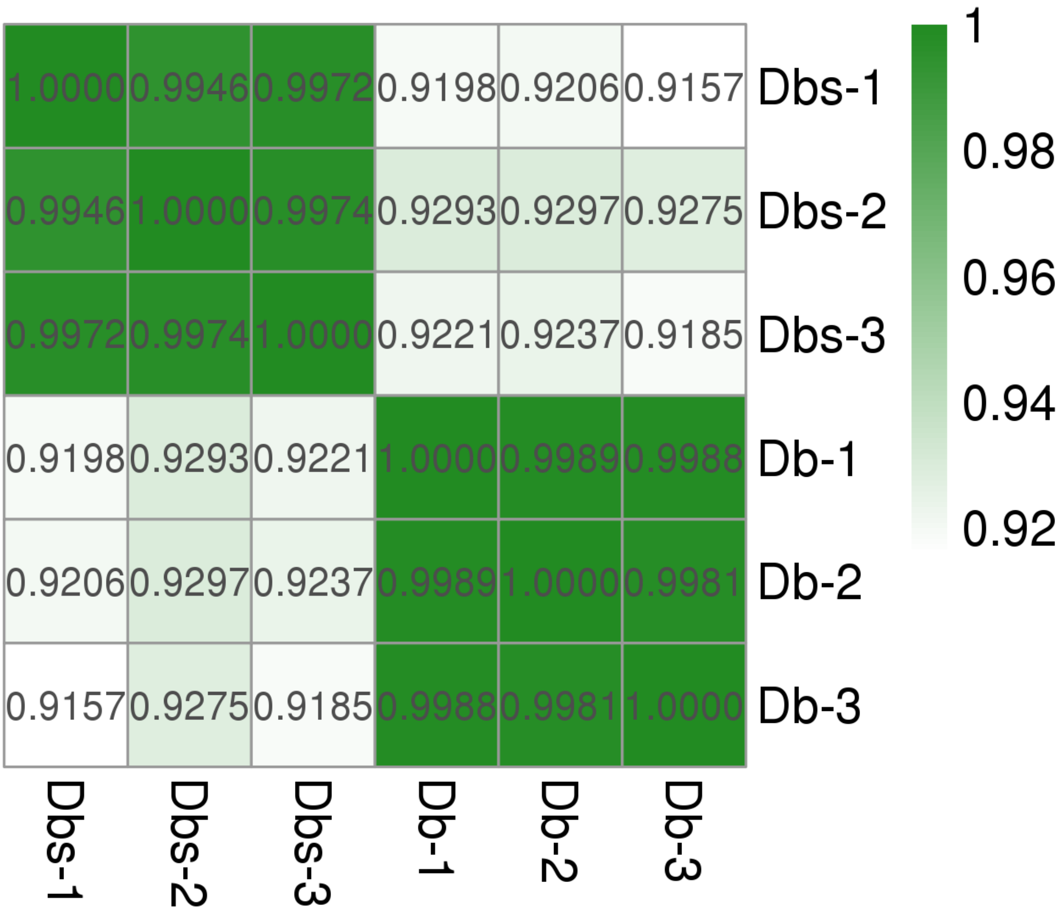

Fig.S2 Samplecorrelationheatmap

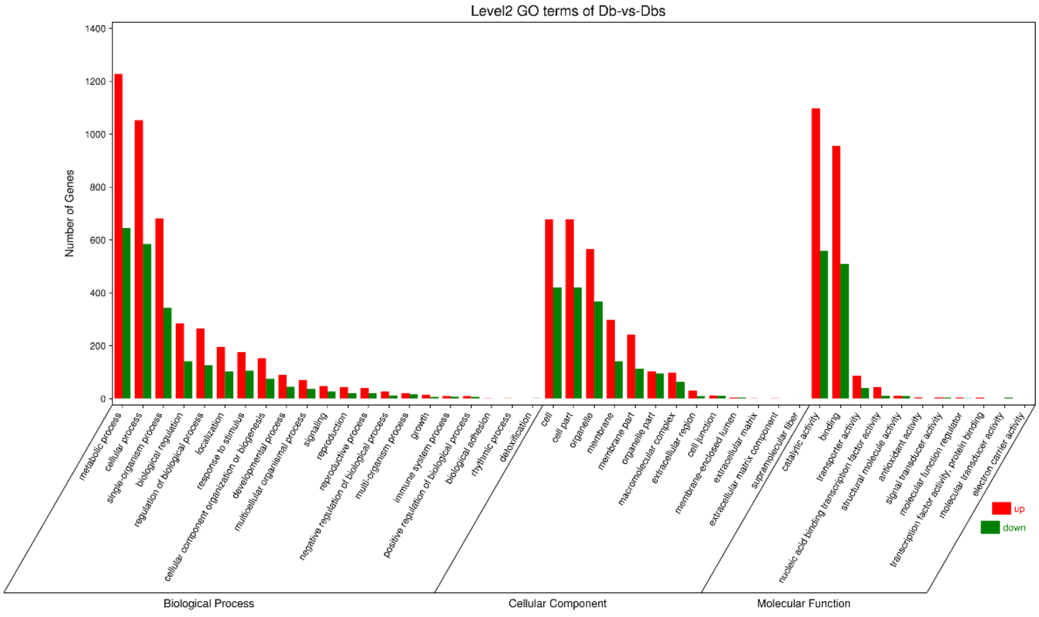

Fig.S3 GOenrichmentanalysisresults

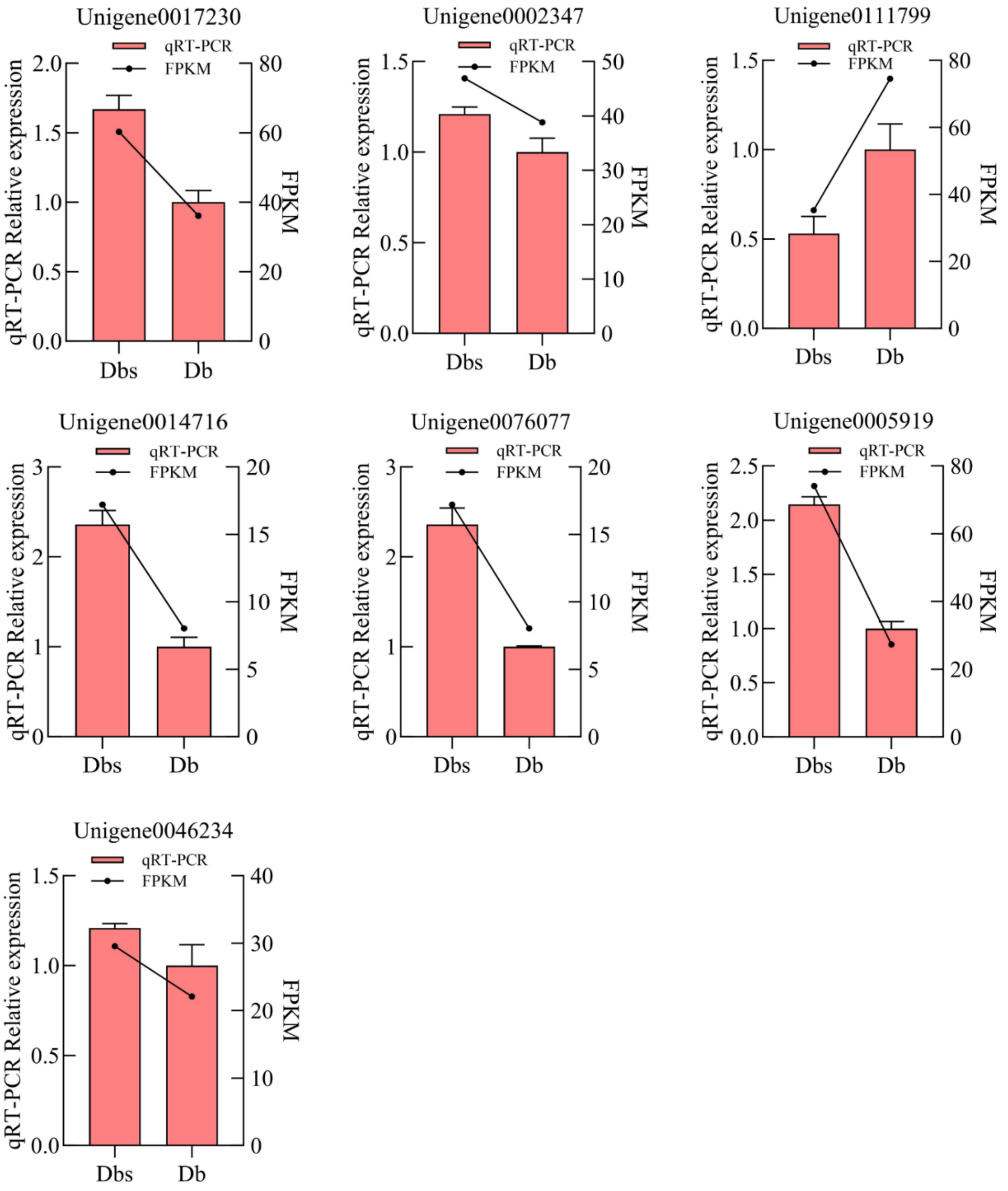

Fig. S4 Validation results of transcriptome sequencing by qRT-PCR
